# Assessing Psychiatric Evaluations in Premature Thelarche and Idiopathic Central Precocious Puberty Cases: Exploring Depression, Anxiety, Quality of Life, and Coping Challenges

**DOI:** 10.7759/cureus.68123

**Published:** 2024-08-29

**Authors:** Zeynep Donbaloglu, Recep Bostan

**Affiliations:** 1 Department of Pediatric Endocrinology, Kepez State Hospital, Antalya, TUR; 2 Department of Child and Adolescent Psychiatry, Kepez State Hospital, Antalya, TUR

**Keywords:** anxiety, depression, quality-of-life, psychiatric disorders, premature telarche, central precocious puberty

## Abstract

Background: The physical and hormonal changes associated with central precocious puberty (CPP) and premature thelarche (PT) can be a significant source of stress for young girls, who may not be developmentally prepared to understand these changes. This study aims to investigate the presence of psychiatric disorders, assess depression, anxiety, coping difficulties, and the quality of life in girls diagnosed with PT, and compare them with idiopathic CPP (ICPP).

Method: A total of 50 participants aged between 6-9 years (PT=33, ICPP=17) between July 2023 and December 2023 were included in the study. Both groups were evaluated by pediatric psychiatrists at a median of four months after diagnosis with ICPP or PT.

Results: The PT group (n=33) had a mean age of 7.81 ± 0.76 years, while the ICPP group (n=17) had a mean age of 8.15 ± 0.35 years (P=0.091). In the PT group, 90.9% were at Tanner stage 2 and 9.1% were at Tanner stage 3 of pubertal development. In the ICPP group, 35.2% were at Tanner stage 2, 58.8% were at Tanner stage 3, and 5.8% were at Tanner stage 4. As expected, compared to the PT group, the ICPP group had taller heights, higher body weights, higher basal and stimulated luteinizing hormone (LH) levels, and elevated follicle-stimulating hormone (FSH) and estradiol (E2) levels (p<0.05).

The total Revised Child Anxiety and Depression Scale (RCADS) score was higher in the PT group compared to the ICPP group (P=0.003). The physical Pediatric Quality of Life (PedsQL) score, psychosocial PedsQL score, and total PedsQL scores were all significantly lower in the PT group (P=0.034, P=0.016, P=0.019, respectively). The total Strengths and Difficulties Questionnaire (SDQ) score, used to evaluate difficulties in coping with challenges, was statistically similar in both groups (P=0.063). Subjects with higher anxiety and depression scores had lower quality of life (P<0.001; R=-0.639). There was also a statistically significant positive correlation between RCADS scores and SDQ scores (P<0.001; R=0.648). We did not find a significant correlation between RCADS scores and hormonal profiles or age.

Conclusion: Despite the hypothalamic-pituitary-gonadal axis not being active, we believe that PT cases may experience psychological impacts due to body changes. While PT cases may not necessitate medical intervention from an endocrinological perspective, addressing their psychological needs can contribute to a healthier overall process for these individuals. The study's findings highlight the need for healthcare providers to monitor the psychological status of PT patients alongside their physical health. Larger-scale studies including control groups are needed to clearly evaluate the relationship between body changes and psychological symptoms.

## Introduction

Puberty is marked by the pulsatile release of gonadotropin-releasing hormone (GnRH) due to the activation of the hypothalamic-pituitary-gonadal (HPG) axis, leading to the development of secondary sexual characteristics [1,2]. Central precocious puberty (CPP) is characterized by the early onset of these traits before the age of eight in girls and before the age of nine in boys, resulting from the premature activation of the HPG axis [1-3]. Idiopathic CPP (ICPP) is diagnosed only after excluding all other possible organic causes [2,3]. Premature thelarche (PT) involves isolated breast development without other clinical signs. In PT cases, unlike in CPP, growth rates are normal, bone age is not advanced, and basal gonadotropin and estradiol levels remain prepubertal [1,4]. Differentiating between these conditions requires clinical assessments, including bone age evaluation and measurements of basal and stimulated gonadotropin levels. The primary treatment for CPP involves the use of GnRH analogs (GnRHa) to delay further pubertal development by suppressing the HPG axis. This intervention is crucial for preserving the potential for normal adult height and for mitigating the psychosocial challenges associated with early puberty and menarche. In contrast, PT cases typically only require clinical follow-up without the need for GnRHa treatment [1].

The physical and hormonal changes associated with CPP and PT can be a significant source of stress for young girls, who may not be developmentally prepared to understand these changes. They might feel different from their peers due to their physical changes, experience embarrassment because of noticeable breast development, and feel restricted in their clothing choices [5-7]. Studies particularly highlight that girls with CPP often experience emotional instability and irritability. These challenges can lead to increased social isolation and withdrawal. Additionally, they may face more bullying and experience feelings of insecurity, disappointment, or shame. These emotional difficulties can negatively impact their relationships with both peers and family members [8,9]. There is limited research on the psychological impact of PT cases. These girls, like those with CPP, show early signs of puberty and must frequently visit hospitals and undergo numerous diagnostic tests. They are monitored without treatment due to the lack of HPG axis activation. However, the psychological impact of coping with physical changes and managing daily life with this condition, even without hormonal activation, remains poorly understood.

This study aims to evaluate clinical and laboratory findings, investigate the presence of psychiatric disorders, assess depression, anxiety, and coping difficulties, and evaluate the quality of life in girls diagnosed with PT compared to those with ICPP.

## Materials and methods

Design

This study was structured as a cross-sectional investigation. Between June 2023 and December 2023, a total of 68 Turkish female patients with suspected precocious puberty presented to the pediatric endocrinology outpatient clinic at the Kepez State Hospital, Antalya, Türkiye, and they were all evaluated by the same pediatric endocrinologist. Among them, 15 patients were determined to have age-appropriate pubertal development. Of the remaining cases, 34 were diagnosed with PT and 19 were diagnosed with CPP. One CPP case, which exhibited detectable organic lesions on magnetic resonance imaging (MRI) scans of the pituitary gland, was excluded to prevent potential physical and emotional impacts associated with such conditions and to maintain sample homogeneity. Additionally, another CPP case was omitted from the study due to the presence of concomitant chronic illnesses and mild mental retardation. Finally, one PT case was excluded from the study due to differing sociodemographic characteristics compared to the general group (being an adopted child with distinct familial properties). Consequently, the final cohort consisted of 50 girls diagnosed with ICPP (n=17) and PT (n=33). None of the included cases presented with additional chronic illnesses or were taking medications known to affect psychological status, puberty, and growth. Furthermore, their sociocultural levels were similar.

Operational definitions

Patients diagnosed with ICPP met the following criteria: (i) the onset of breast development before the age of eight years, (ii) a basal luteinizing hormone (LH) level above 1.0 IU/L or a peak LH level greater than 5 IU/L in response to the LH-releasing hormone stimulation test, (iii) accelerated growth and a bone age at least one year ahead of chronological age, and (iv) no hypothalamic-pituitary lesions as confirmed by MRI scans [1]. Subjects meeting these criteria received subcutaneous injections of 3.75 mg of GnRHa (leuprolide acetate, Lucrin Depot®) every 28 days. Those who showed breast development before the age of eight years but had suppressed basal LH levels and a peak LH level below 5 IU/L in the LH-RH stimulation test were classified as having PT [1].

Clinical and laboratory measures

Height, weight, and body mass index (BMI) standard deviation scores (SDS) were calculated using reference values specific to Turkish children [10]. BMI was calculated as the ratio of weight to height squared (kg/m²). The pubertal staging was evaluated based on the criteria established by Marshall and Tanner [11]. LH and follicle-stimulating hormone (FSH) levels were measured in the early morning at 08:00-09:00 am using chemiluminescence immunoassay, while estradiol (E2) levels were determined using the electrochemiluminescence immunoassay method. All hormone assessments were conducted by Roche in Mannheim, Germany.

Psychiatric evaluation

A child and adolescent psychiatrist with specialized expertise conducted the psychiatric assessments. The cases were monitored for at least three to six months after being diagnosed with PT or ICPP, and psychiatric interviews were conducted following this period. The participants' sociodemographic characteristics, such as parental education level and family income, were noted, and the assessments given below were administered to all participants.

Revised Child Anxiety and Depression Scale (RCADS)

This inventory was developed to assess depression and anxiety in children and adolescents and has been subsequently validated and found applicable for Turkish children through validation and reliability studies [12,13]. The scale consists of 47 items, with four options for each question. Participants mark each item as 0 if it is "never true," 1 if "sometimes true," 2 if "often true," and 3 if "always true," resulting in a total score ranging from 0 to 141. A T-score of 65 or above is considered clinically significant. The inventory includes subscales for various disorders: seven items for separation anxiety disorder (SAD), nine items for social phobia (SP), six items for generalized anxiety disorder (GAD), nine items for panic disorder (PD), six items for obsessive-compulsive disorder (OCD), and 10 items for major depressive disorder (MDD). In the Turkish adaptation, cutoff scores are 5.5 for SAD, 9.5 for SP, 7.5 for GAD, 6.5 for PD, 7.5 for OCD, and 11.5 for MDD. The Turkish version demonstrates high internal consistency with a coefficient of 0.95 and subscale coefficients ranging from 0.75 to 0.86 [13].

Strengths and Difficulties Questionnaire (SDQ)

This inventory was used to assess behavioral and emotional issues, with parents completing the assessment [14]. The questionnaire includes 25 items divided into five subscales, each with five items. The SDQ measures four problem areas: conduct problems (CP), hyperactivity-inattention problems (HA), emotional symptoms (EP), and peer relationship problems (PP). Additionally, there is a subscale for personal strengths that assesses prosocial (PS) behaviors such as sharing and consideration of others' feelings. A total difficulties score is calculated by summing the scores of the four problem subscales, excluding the PS behavior subscale. Higher scores on the CP, HA, EP, and PP subscales and the total difficulties score indicate having more problems and difficulties in related areas. On the other hand, higher scores on the PS behavior subscale indicate strengths. The Turkish adaptation also has demonstrated consistency and reliability [15].

Pediatric Quality of Life Inventory (PedsQL)

This inventory was developed to assess health-related quality of life (HRQoL) in children and adolescents. It includes 23 items evaluating physical and psychosocial functioning, rated on a five-point Likert-type scale (0 = never, 1 = rarely, 2 = sometimes, 3 = often, 4 = always). Scores are transformed linearly to a range between 0 and 100 points, with higher scores reflecting higher HRQoL. In the Turkish reliability and validity study for children aged 5-7 years, the internal consistency reliability alpha coefficient for the total scale score was found to be 0.80 [16], and for children aged 8-12 years, it was 0.86 [17].

Statistical analysis

Statistical analyses of our study were conducted using IBM SPSS Statistics for Windows, Version 23.0 (Released 2015; IBM Corp., Armonk, New York, United States). The normality of continuous variables was assessed using the Shapiro-Wilk test. If the variables were continuous and normally distributed, the results were expressed as mean and standard deviation. A comparison of variables within this group was performed using the T-test (age, height, BMI). If the continuous variables did not follow a normal distribution, the results were presented as the median and interquartile range (IQR), representing the range between the 25th and 75th percentiles. A comparison of variables within this group was conducted using the Mann-Whitney U test (weight, weight SDS, height SDS, BMI SDS, LH, FSH, E2, and scores of all psychological measures). Categorical variables were presented as frequencies and percentages. Pearson's chi-square and Fisher's exact tests were used to compare categorical variables (Tanner stage, rates of RCADS subscales). Correlation analysis was carried out using Spearman's rho test to evaluate relationships between variables. The strength of the relationship was interpreted based on the following r values: very weak (< 0.25), weak (0.26 to 0.49), medium (0.50 to 0.69), high (0.70 to 0.89), and very high (0.90 to 1.0). Statistical significance was considered at p < 0.05.

Ethics

This study was conducted in compliance with the principles outlined in the Declaration of Helsinki. The research protocol was reviewed and approved by the Akdeniz University Ethics Committee (approval number: KAEK-461 dated 07.06.2023), ensuring that the rights, safety, and well-being of the participants were safeguarded. Informed consent was obtained from all participants and their guardians prior to inclusion in the study. Confidentiality of the participants’ data was maintained throughout the research process, and all procedures adhered to ethical standards to ensure the integrity and ethical conduct of the study.

## Results

The PT group (n=33) had a mean age of 7.81 ± 0.76 years, while the ICPP group (n=17) had a mean age of 8.15 ± 0.35 years (P=0.091). In the PT group, 30 (90.9%) cases were at Tanner stage 2, and three (9.1%) cases were at Tanner stage 3 of pubertal development. In the ICPP group, six (35.2%) cases were at Tanner stage 2, 10 (58.8%) cases were at Tanner stage 3, and one (5.8%) case was at Tanner stage 4. As expected, compared to the PT group, the ICPP group had taller heights, higher body weights, higher basal and stimulated LH levels, and elevated FSH and E2 levels (p<0.05) (Table 1).

**Table 1:** Clinical properties of the cases Data are expressed as mean±SD, median (interquartile range), or number (percentage), as marked. *Statistically significant comparison (p<0.05); ^a^ T-test; ^b^ Mann-Whitney U test; ^c^ Fisher's Exact test PT: premature thelarche; ICPP: idiopathic central precocious puberty; SDS: standard deviation score; BMI: body mass index, LH: luteinizing hormone; FSH: follicle-stimulating hormone; E2: estradiol; LHRH: luteinizing hormone-releasing hormone.

Variable	PT (n=33)	ICPP (n=17)	p-value
Age (year), mean±SD	7.81 ± 0.76	8.15 ± 0.35	0.091^a^
Time after diagnosis (month), median (IQR)	4 (2)	4 (1.25)	0.227^b^
Tanner Stage: 2/3/4, n (%)	30 (90.9%)/3 (9.1%)/0 (0%)	6 (35.2 %)/10 (58.8%)/1 (5.8%)	<0.001^c^*
Weight (kg), median (IQR)	28.7 (9)	35 (13.3)	0.030^b^*
Weight SDS, median (IQR)	0.83 (2.04)	1.34 (1.58)	0.224^b^
Height (cm), mean±SD	132.4± 5.1	140.7 ± 6.37	<0.001^c^*
Height SDS, median (IQR)	1.34 (1.49)	1.92 (0.96)	0.024^b^*
BMI (kg/m^2^), mean±SD	17.1 ± 3.44	17.6 ± 2.8	0.467^a^
BMI SDS, median (IQR)	0.30 (2.13)	0.73 (1.64)	0.984^b^
LH (mIU/mL), median (IQR)	0.1 (0.1)	0.74 (0.9)	<0.001^b^*
LH (peak on LHRH test, mIU/mL), median (IQR)	1.39 (1.82)	10.5 (11.7)	<0.001^b^*
FSH (mIU/mL), median (IQR)	1.52 (1.93)	3.59 (4.79)	0.009^b^*
E2 (pg/mL), median (IQR)	20.3 (6.3)	26.9 (40.3)	0.026^b^*

**Figure 1:**
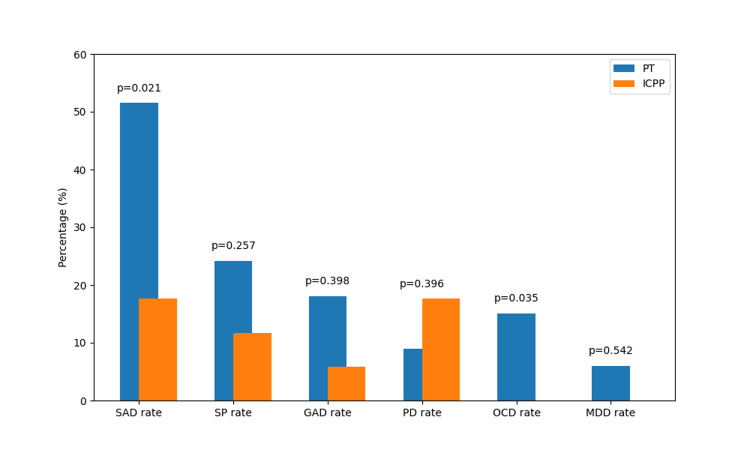
Rates of subgroups of the Revised Child Anxiety and Depression Scale in PT and ICPP cases Comparisons were performed with Fisher's Exact test. PT: premature thelarche; ICPP: idiopathic central precocious puberty; SAD: separation anxiety disorder; SP: social phobia; GAD: generalized anxiety disorder; PD: panic disorder; OCD: obsessive-compulsive disorder; MCD: major depressive disorder.

Both groups were evaluated by a pediatric psychiatrist at a median of four months after diagnosis with ICPP or PT. When evaluating the anxiety and depression levels of the subjects using RCADS scores, two (6%) cases in the PT group had scores above 65, the threshold considered clinically significant for a disorder, while no cases in the ICPP group exceeded this threshold. Additionally, the total RCADS score was statistically significantly higher in the PT group compared to the ICPP group (P=0.003). Furthermore, when examining the RCADS subscale scores, the SAD, OCD, and MDD scores were statistically significantly higher in the PT group (P=0.007, P=0.024, P=0.040, respectively). SAD was quite prevalent, observed in 17 (51.5%) of the PT cases (Table 2, Figure 1).

**Table 2:** Psychiatric evaluations of the cases: Revised Child Anxiety and Depression Scale Data are expressed as median (interquartile range) and number (percentage) *Statistically significant comparison (p<0.05); ^a ^Mann-Whitney U test, ^b ^Fisher's Exact test. PT: premature thelarche; ICPP: idiopathic central precocious puberty; RCADS: Revised Child Anxiety and Depression Scale; SAD: separation anxiety disorder; SP: social phobia; GAD: generalized anxiety disorder; PD: panic disorder; OCD: obsessive-compulsive disorder; MCD: major depressive disorder.

Variable	PT (n=33)	ICPP (n=17)	p-value
RCADS score (total)	24 (26)	16 (17)	0.030^a^*
SAD score	6 (6)	3 (3.5)	0.007^a^*
SAD rate, n (%)	17 (51.5 %)	3 (17.6 %)	0.021^b^*
SP score	6 (5.5)	4 (4)	0.092^a^
SP rate, n (%)	8 (24.2 %)	2 (11.7 %)	0.257^b^
GAD score	4 (3.5)	3 (3)	0.215^a^
GAD rate, n (%)	6 (18.1 %)	1 (5.8 %)	0.398^b^
PD score	2 (2.5)	2 (3)	0.624^a^
PD rate, n (%)	3 (9.0 %)	3 (17.6 %)	0.396^b^
OCD score	3 (4)	1 (3.5)	0.024^a^*
OCD rate, n (%)	5 (15.1 %)	0	0.035^b^*
MDD score	4 (6)	1 (3.5)	0.040^a^*
MDD rate, n (%)	2 (6 %)	0	0.542^b^

To assess the quality of life, the physical PedsQL score, psychosocial PedsQL score, and total PedsQL score were all significantly lower in the PT group (P=0.034, P=0.016, P=0.019, respectively). The total SDQ score, used to evaluate difficulties in coping with challenges, was higher in the PT group, but this difference was not statistically significant (P=0.063). However, in the SDQ subscales, scores related to conduct problems were higher in the PT group (P=0.043) (Table 3).

**Table 3:** Psychiatric evaluations of the cases: the Pediatric Quality of Life and The Strengths and Difficulties Questionnaire Data are expressed as median (Q1 - Q3 quartiles). *Statistically significant comparison (p<0.05) with Mann-Whitney U test. PT: premature telarche; ICPP: idiopathic central precocious puberty; PedsQL: Pediatric Quality of Life; SDQ: Strengths and Difficulties Questionnaire; CP: conduct problems; HA: hyperactivity; EP: emotional problems; PP: peer problems; PS: prosocial.

Variable	PT (n=33)	ICPP (n=17)	p-value
Physical PedsQL score	78.1 (62.5 - 89)	87.5 (73.4 - 96.8)	0.034*
Psychosocial PedsQL score	81.6 (70 - 91.6)	90 (79.1 - 95.8)	0.016*
Total PedsQL score	80.4 (71.1 - 88)	86.9 (80.4 - 95)	0.019*
SDQ - Total difficulties score	9 (6.5 - 12)	6 (3 - 6)	0.063
CP scale	1 (1 - 2)	0 (0 - 1.5)	0.043*
HA scale	5 (3 - 6)	4 (1.5 - 5.5)	0.178
EP scale	1 (0 - 3)	1 (0 - 2)	0.719
PP scale	2 (1 - 2)	1 (0 - 2)	0.380
PS scale	9 (7.5 - 10)	8 (7.5 - 10)	0.760
Internalising score	3 (1.5 - 5 )	2 (1 - 4)	0.294
Externalising score	6 (4 - 7)	5 (1.5 - 6)	0.055

In the correlation analyses of the entire group, we observed that subjects with higher anxiety and depression scores had lower quality of life (P<0.001; R=-0.639). There was also a statistically significant positive correlation between RCADS scores and SDQ scores (P<0.001; R=0.648). We did not find a significant correlation between RCADS scores and hormonal profiles or age. SDQ scores showed a negative correlation with the total PedsQL score (P<0.001; R=-0.632). While SDQ scores did not correlate with LH levels, they were positively correlated with FSH and E2 levels (P=0.017; R=0.344 and P=0.007; R=0.383) (Table 4).

**Table 4:** Correlational analyses between clinical parameters and psychiatric evaluation scores *Statistically significant correlation (p<0.05) with Spearman's rho test; R= correlational coefficient. RCADS: Revised Child Anxiety and Depression Scale; PedsQL: Pediatric Quality of Life Inventory; SDQ: Strengths and Difficulties Questionnaire; CP: conduct problems; HA: hyperactivity; EP: emotional problems; PP: peer problems; PS: prosocial. LH: luteinizing hormone; FSH: follicle-stimulating hormone; E2: estradiol; LHRH: luteinizing hormone-releasing hormone.

	RCADS		Total PedsQL	
	P	R	P	R
RCADS	-	-	<0.001*	-0.639
Total PedsQL	<0.001*	-0.639	-	-
SDQ - Total difficulties score	<0.001*	0.648	<0.001*	0.632
SDQ - Internalising score	<0.001*	0.585	<0.001*	-0.587
SDQ - Externalising score	<0.001*	0.557	<0.001*	0.519
CP scale	0.004*	0.400	0.001*	-0.449
HA scale	0.001*	0.461	0.002*	0.424
EP scale	<0.001*	0.641	<0.001*	-0.554
PP scale	0.025*	0.316	0.008*	-0.371
PS scale	0.990	0.002	0.317	0.144
Age	0.664	0.061	0.417	0.117
LH basal	0.601	0.077	0.967	-0.006
FSH basal	0.071	0.263	0.097	-0.241
E2 basal	0.293	0.155	0.368	-0.133
LH peak at LHRH	0.089	-0.425	0.111	0.401

## Discussion

In our study, we observed higher anxiety and depression scores, along with a lower quality of life scores in PT cases compared to ICPP cases when evaluated three months post diagnosis. This study sheds light on an important aspect of PT cases, highlighting the psychological challenges they face despite the absence of hormonal activation.

Pubertal timing significantly influences adolescents' mental health and psychosocial well-being. Early development of secondary sexual characteristics can heighten emotional struggles as individuals face new pressures and societal expectations before being emotionally ready to handle them. These physical changes may lead to uncomfortable social situations, fears of rejection, or actual experiences of it. Studies have shown that girls, in particular, may experience concerns about differences in breast development, negatively affecting self-esteem and fostering feelings of unhealthiness, loneliness, and even depression [18,19].

Information regarding the psychological aspects of precocious puberty in girls remains inconsistent and limited. GnRHa is widely regarded as the primary treatment for CPP, effectively suppressing the HPG axis [20]. While the main objective of treating CPP is to maintain adult height potential, addressing the psychological distress linked with early pubertal development is frequently highlighted as a secondary concern [1]. In ICPP cases, GnRHa treatment suppresses the HPG axis, aiming to control both physical and emotional changes of puberty. However, in PT cases, where the HPG axis is already suppressed, treatment is typically not required. Therefore, the higher psychological assessment scores observed in PT cases cannot be solely attributed to hormonal profiles. Consistent with this, in our study, correlation analyses revealed no significant relationship between hormone levels and RCADS or PedQL scores. However, the medical treatment provided to the ICPP group for secondary pubertal characteristics may offer morale and motivation, as it presents a solution to their condition alongside hormonal suppression. Conversely, the absence of medical intervention in PT cases, despite the presence of secondary pubertal characteristics, may heighten anxiety levels and lead to a decline in quality of life. Furthermore, the correlation between anxiety, depression, and lower quality of life underscores the interconnectedness of these factors in influencing the overall well-being of PT patients.

In a study assessing the QoL among CPP, PT, and control groups irrespective of age, no statistically significant differences were observed in scale scores. However, within the 8-12 age bracket, the emotional functioning subscale of QoL was notably lower in the CPP group compared to both the PT and control groups [21]. Given the cross-sectional design of our study and the absence of extended follow-up periods, we were unable to evaluate the potential long-term psychological ramifications of GnRHa therapy as our cases progressed into this age range.

Literature reviews indicate that girls with CPP undergoing GnRHa therapy, compared to healthy controls, do not report significant differences in mean depression scores between patients and controls [22]. Similarly, Melissa et al. observed normal range scores on the Children's Depression Inventory and Child Behavior Checklist (CBCL) at baseline when assessing the psychological aspects of girls with CPP, premature adrenarche, and early normal puberty [23]. Additionally, another study involving 33 girls with ICPP and healthy controls revealed no significant differences in the anxious/depressed subscale scores of the CBCL [6]. In our study, none of the CPP cases exhibited elevated MDD scores, whereas 6% of the PT cases did. Additionally, in approximately one out of every two cases of premature PT, the SAD score was found to be elevated. A cohort study involving 138 cases found that early-maturing girls experienced significantly greater social anxiety compared to on-time girls and early-maturing boys [24]. However, it's worth noting that this study's cases were evaluated between ages 12 and 17, with 68 being girls, among whom 10 were classified as early-maturing. The assessment of pubertal timing in this study relied on self-reported pubertal status, making it unclear how many of these cases had CPP or had previously used GnRHa [24].

In the current case, the SDQ scores were normal between the groups, except for the conduct problems subscale, which was higher in the PT group. Internalizing and externalizing symptoms were also similar between the two groups. Prosocial behavior traits were also similar, with both groups exhibiting high scores indicative of positive prosocial behaviors. Prosocial behavior is characterized by a child's ability to interact well with peers and engage in actions that benefit those around them [25]. One study suggested that puberty might enhance prosocial behaviors by increasing emotional responsiveness to others in need. Additionally, high prosocial scores may be linked to psychological maturity [26]. This finding could correlate with the development of secondary sexual characteristics and physical maturation in both ICPP and PT cases, fostering a sense of growth and facilitating the adoption of more adult-like behavior patterns.

Our study has certain limitations. First, the absence of a control group prevents us from making clearer comparisons with healthy peers. Second, since our study is cross-sectional, it does not include long-term data for the cases. Third, the number of patients in our study group is too small and restricted to Turkish children, which hinders the generalization of the results to the general population. Fourthly, psychological evaluations are based on inventories. For instance, the assessment of social anxiety using the RCADS does not encompass physiological indicators (such as sweating) or behavioral responses (like avoidance).

## Conclusions

In our study, based on survey assessments, we observed that PT cases have higher anxiety and depression scores and lower quality of life scores compared to ICPP cases. Despite the HPG axis not being active, we believe that PT cases may experience psychological impacts due to body changes. While PT cases may not necessitate medical intervention from an endocrinological perspective, addressing their psychological needs can contribute to a healthier overall process for these individuals. The study's findings highlight the need for healthcare providers to monitor the psychological status of PT patients alongside their physical health. Larger-scale studies including control groups are needed to clearly evaluate relationship between body changes and psychological symptoms. This study may serve as a preliminary investigation and its findings are limited in scope, necessitating further research to generalize the results to the broader population.
